# Effects of Curcumin and Its Different Formulations in Preclinical and Clinical Studies of Peripheral Neuropathic and Postoperative Pain: A Comprehensive Review

**DOI:** 10.3390/ijms22094666

**Published:** 2021-04-28

**Authors:** Paramita Basu, Camelia Maier, Arpita Basu

**Affiliations:** 1 Pittsburgh Center for Pain Research, and the Pittsburgh Project to End Opioid Misuse, Department of Anesthesiology & Perioperative Medicine, University of Pittsburgh School of Medicine, Pittsburgh, PA 15213, USA; PAB171@pitt.edu; 2 Department of Biology, Texas Woman’s University, Denton, TX 76204-5799, USA; cmaier@twu.edu; 3 Department of Kinesiology and Nutrition Sciences, School of Integrated Health Sciences, University of Nevada, Las Vegas, NV 89154, USA

**Keywords:** curcumin, curcumin formulations, diabetes, peripheral neuropathy, postoperative pain, preemptive analgesia

## Abstract

Lesion or disease of the somatosensory system leads to the development of neuropathic pain. Peripheral neuropathic pain encompasses damage or injury of the peripheral nervous system. On the other hand, 10–15% of individuals suffer from acute postoperative pain followed by persistent pain after undergoing surgeries. Antidepressants, anticonvulsants, baclofen, and clonidine are used to treat peripheral neuropathy, whereas opioids are used to treat postoperative pain. The negative effects associated with these drugs emphasize the search for alternative therapeutics with better efficacy and fewer side effects. Curcumin, a polyphenol isolated from the roots of *Curcuma longa,* possesses antibacterial, antioxidant, and anti-inflammatory properties. Furthermore, the low bioavailability and fast metabolism of curcumin have led to the advent of various curcumin formulations. The present review provides a comprehensive analysis on the effects of curcumin and its formulations in preclinical and clinical studies of neuropathic and postoperative pain. Based on the positive outcomes from both preclinical and clinical studies, curcumin holds the promise of mitigating or preventing neuropathic and postoperative pain conditions. However, more clinical studies with improved curcumin formulations are required to involve its use as adjuvant to neuropathic and postoperative drugs.

## 1. Introduction

Neuropathic pain has been defined as a process occurring after a primary lesion or the disease of the somatosensory nervous system [1]. Based on either clinical examination or self-reporting, the prevalence of neuropathic pain is 9.8% and 12.4%, respectively, in the United States. However, due to differences in defining neuropathic pain, and employing different epidemiological assessment methods, it is difficult to provide the accurate estimate of neuropathic pain [2]. Peripheral neuropathic pain refers to damage or injury to the peripheral nerves [3]. According to the Special Interest Group on Neuropathic Pain, gabapentinoids, tricyclic antidepressants, and selective serotonin–norepinephrine reuptake inhibitors have been identified as the first-line drugs for neuropathic pain, whereas lidocaine, capsaicin, and tramadol are considered second-line drugs. Opioids such as morphine, oxycodone, and botulinum toxin-A are included as third-line treatments for peripheral neuropathic pain [4]. However, these drugs are accompanied by several side effects that limit their use in preventing or treating neuropathic pain [4].

On the other hand, acute postoperative pain is followed by persistent pain in 10–15% individuals undergoing surgeries, such as breast, bypass, and thoracic surgery, coronary, groin hernia repair, and leg amputation [5]. Opioids are effectively used to treat postoperative pain [6]. However, prolonged use of opioids for chronic postoperative pain treatment is less efficacious and is associated with major side effects, such as addiction, dependence, liability, and opioid-induced hyperalgesia, nausea, vomiting, pruritus, and bowel motility reduction leading to ileus and constipation [7,8,9,10].

Taken together, the negative side effects associated with neuropathic and postoperative pain treatment reinforce the recommendations of evidence-based alternative therapeutics to treat these aforementioned pain conditions. Natural products, including herbals, flavonoids, and polyphenols, remain one of the most promising treatments for neuropathic [11,12,13,14,15,16] and postoperative pain [17,18]. Curcumin, also known as diferuloylmethane, is a polyphenolic compound present in the rhizomes of *Curcuma longa* (syn. *C. domestica* Valeton and *C. brog* Valeton) (*Zingiberaceae*) used as a culinary spice in curry powder [19]. The antioxidant and anti-inflammatory properties of curcumin [20,21] could be attributed to its antinociceptive activity against different pain conditions, including peripheral neuropathic, inflammatory, postoperative, burn pain, and wound healing [22,23,24,25], as well as its use as an oral supplement in the treatment of various inflammatory medical conditions, such as alopecia [26].

Different curcumin formulations have been employed in order to improve the bioavailability mainly by providing protection against chemical degradation of curcumin [27,28,29,30,31,32,33,34]. These formulations include encapsulation technologies of curcumin into nanoparticles or microparticles and introduced in food or supplemental products [35]. Furthermore, the different colloidal particles that are used to incorporate curcumin include micellar aggregates, biopolymer particles, emulsion droplets, liposomes, and solid lipid particles [36].

The present review provides insights into the analgesic effects of curcumin and its formulations in neuropathic and postoperative pain conditions and explores its limitations in preclinical and clinical studies. To the best of our knowledge, this is the first comprehensive review that provides in-depth insights into the effects of curcumin and its different formulations on behavioral, electrophysiological, and molecular aspects of both peripheral neuropathic and postoperative pain.

## 2. Materials and Methods

The literature search mainly focused on the effects of curcumin and its different formulations on preclinical studies, especially in rodent models of peripheral neuropathic and postoperative pain conditions. However, a part of the review also presented the effects of curcumin and its formulations in clinical studies of neuropathic and postoperative pain. Outcome measures of interest were different behavioral modalities, such as mechanical, thermal (heat and cold), motor coordination, electrophysiological parameters, and molecular markers of antioxidative enzymes, reactive oxygen species (ROS), inflammation, and many more. All searches were conducted in PubMed and Google Scholar, and articles published between 2010 and 2021 were selected for the review. Key search terms were “curcumin”, “curcumin formulations”, “curcumin and peripheral neuropathic pain”, “curcumin and postoperative pain”, “curcumin bioavailability”, “curcumin and neuropathic and clinical studies”, and “curcumin and postoperative pain and clinical studies”.

## 3. Structure, Source, Metabolism, and Bioavailability of Curcumin

### 3.1. Chemical Structure

**Figure 1:**
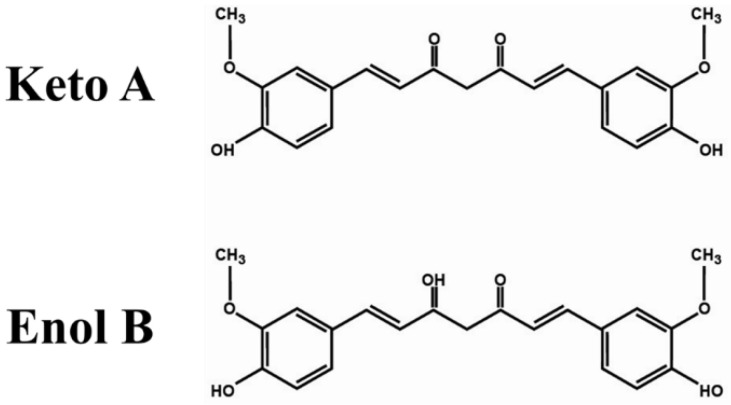
Keto A and enol B forms of curcumin.

Structurally, the chemical skeleton of curcumin [1,7-bis(4-hydroxy-3-methoxyphenyl)-1,6-heptadiene-3,5-dione] possesses different functional moieties that are bonded to two phenol rings [37]. Two sets of α- and β-unsaturated carbonyl moieties link the phenol rings [38,39], responsible for reaction with biological nucleophiles by employing Michael addition and C-C adducts [40,41]. Furthermore, the chemical structure of curcumin contains two methoxy aryl moieties at the ortho positions, a hydroxy substituent, and conjugated β-diketone moieties [41]. Curcumin exists in two different tautomeric forms: 1,3-diketo and enol forms [42,43] (Figure 1).

### 3.2. Source and Metabolism

**Figure 2:**
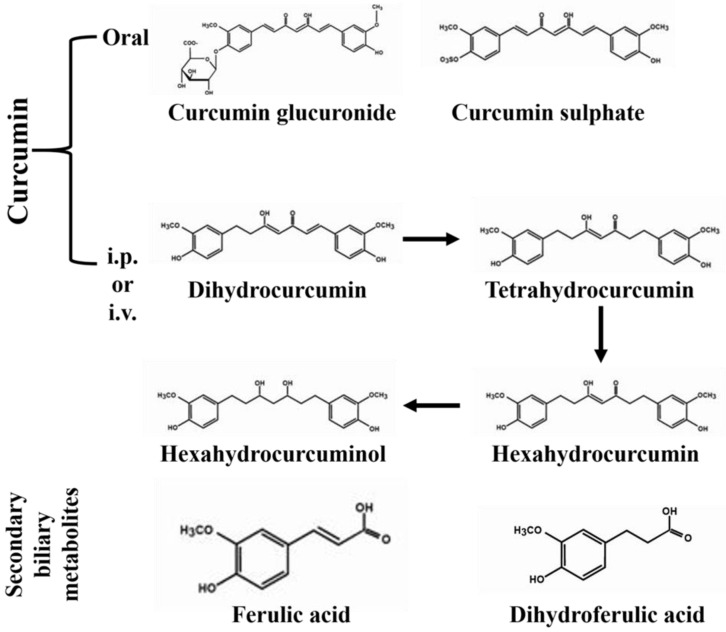
Structures of curcumin metabolites following oral or intraperitoneal (i.p.) or intravenous (i.v.) administration.

The genus *Curcuma* is widely cultivated in tropical and sub-tropical regions of Asia, Australia, and South America [44]. Curcumin is obtained from the tuberous rhizomes of *C. longa*, which is known as “turmeric” worldwide. *C. longa* is widely cultivated in India, China, and Indonesia [19,45]. The active components of *Curcuma* rhizomes involve volatile oils and nonvolatile curcuminoids (curcumin, demethoxycurcumin, bisdemethoxycurcumin), which are nontoxic polyphenolic derivatives with several biological activities [46,47]. Curcumin shows unfavorable pharmacokinetic properties (adsorption, distribution, excretion, and metabolism), insolubility in aqueous solutions, instability in neutral and alkaline pH, as well as sensitivity of both solid and solubilized forms to light [48]. Curcumin metabolism mainly takes place in the liver, but also in the intestine by gut microbiota [49]. It is rapidly metabolized either through the phase II conjugation of curcumin-to-curcumin glucuronide and curcumin sulphate in the intestine and hepatic cytosol or phase I enzymatic reduction of curcumin to dihydrocurcumin, tetrahydrocurcumin, hexahydrocurcumin, and hexahydrocurcuminol in the enterocytes and hepatocytes [50,51,52]. Furthermore, glucuronidation occurs on reduced curcumin, leading to formation of curcumin glucuronide, dihydro-curcumin-glucuronide, tetrahydrocurcumin-glucuronide, and curcumin sulfate [50]. Dihydro-ferulic acid and ferulic acid are also formed as the products of secondary biliary metabolism [53,54] (Figure 2).

### 3.3. Bioavailability

Table 1 summarizes the serum and tissue levels of curcumin in rodents and humans followed by different routes of administration. Despite its efficacy and safety, the poor bioavailability of curcumin undermines its therapeutic potential. Animal studies reported that oral administration [55,56] of curcumin led to its poor absorption, rapid metabolism, and excretion. Oral consumption of curcumin leads to the rapid formation of conjugates, such as curcumin glucuronide and curcumin sulfate in the small intestine, liver, and kidneys. The conjugates undergo rapid excretion in the urine and feces [50,57,58,59,60,61,62]. In humans, curcumin has poor bioavailability, even when administered at a dose of 12 g/day [63]. Moreover, in humans, the oral bioavailability of curcumin is low because of its low absorption in the small intestine coupled to an extensive reduction and conjugation into metabolites in the liver followed by elimination through the gall bladder [54,64].

## 4. Curcumin and Neuropathic Pain—Preclinical Studies

### 4.1. Alcoholic Neuropathy

Chronic alcohol consumption leads to an array of neurological aberrations, including cortical and motility dysfunction, delirium tremens, psychosis, peripheral polyneuropathy, and Wernicke encephalopathy [72]. Direct toxicity to alcohol, family history of alcoholism, malnutrition, and thiamine deficiency are associated with alcoholic neuropathy [73,74,75,76]. However, it is unclear which of the above factors induces neuropathy [77]. Moreover, alcohol promotes oxidative stress by decreasing the endogenous concentrations of antioxidants, such as α-tocopherol, ascorbate, and vitamin E [78], generating ROS and lipid peroxidation [79], and damaging DNA, cellular protein, and other signaling pathways that regulate oxidative stress [78].

Table 2 summarizes the effects of curcumin on alcoholic neuropathy. Curcumin reduced alcohol-induced mechanical and thermal (heat and cold) hypersensitivity, inhibited the reduction in motor nerve conduction velocity (MNCV), reduced inflammatory cytokines, such as tumor necrosis factor alpha (TNF-α) and interleukin-1β (IL-1β), and reduced DNA fragmentation [80]. Curcumin also exerts antioxidant properties by reducing the levels of calcium, neural nitrite, and malondialdehyde (MDA), and restoring glutathione (GSH) levels [80]. Curcumin has also revealed better efficacy in reducing the alcohol-induced behavioral outcomes and inflammatory cytokines when compared to the standard drug α-tocopherol [80]. Treatments with curcumin or sildenafil, a drug that is reported to reduce neuropathic pain in rodent models [81,82], did not reduce the alcohol-induced pain behaviors and did not alter oxidative stress biomarkers [83]. However, combined low doses of curcumin and sildenafil were effective in treating chronic alcohol-induced neuropathic pain. The results confirm that low doses of curcumin and sildenafil might have interacted synergistically to mitigate alcoholic neuropathy [83]. Therefore, the drug–supplement association might provide a therapeutic advantage in treating alcoholic neuropathy under clinical settings.

### 4.2. Chemotherapy-Induced Peripheral Neuropathy (CIPN)

The treatment of cancer with different anticancer agents, including vinca alkaloids, platinum drugs (cisplatin and oxaliplatin), taxanes, and other chemotherapeutic drugs, leads to CIPN, which affects 30–40% of patients [98]. The symptoms of CIPN initiate with the onset of chemotherapy and improve with the completion of the therapy. However, 25–30% patients experience pain or unpleasant paresthesia, which even persists after chemotherapy completion [99]. Moreover, CIPN could potentially lead to a decrease in the dose of chemotherapeutics, change to less effective agents, and even cause cessation of the treatment [100]. In terms of cellular mechanisms, anticancer drugs paclitaxel, vincristine, and oxaliplatin lead to mitochondrial damage of sensory neurons in the dorsal root ganglion (DRG), leading to the increased production of ROS [101,102,103,104]. Chemotherapy leads to the cellular respiration impairment and decreases the production of adenosine triphosphate (ATP). Therefore, promoting mitochondrial respiration and restoring mitochondrial bioenergetics provide protection against CIPN [105,106]. Furthermore, the anticancer drug treatment leads to the reduction in antioxidative enzymes, such as superoxide dismutase (SOD) and catalase (CAT), causing an imbalance between oxidant and antioxidant molecules [101,104,107]. This imbalance promotes the cellular apoptotic pathways, leading to degeneration of peripheral sensory fibers and other inflammatory events [108,109]. Therefore, antioxidant therapy is considered as an effective treatment against CIPN [110].

Table 2 summarizes the effects of curcumin on CIPN. Curcumin improved platinum drug cisplatin or oxaliplatin-induced thermal (heat or cold) and mechanical hypersensitivity [85,86,87], and formalin test [87] in various strains of rodent models. However, Al Moundhri et al. [88] reported that curcumin could not attenuate cisplatin- or oxaliplatin-induced painful behavioral outcomes. The study attributed a few factors, such as low number of animals in each treatment group, administration of low concentration of curcumin, and other unknown factors to this effect [88]. Curcumin also did not exert any impairment of neuromuscular coordination, indicating that curcumin did not alter motor coordination [88].

Electrophysiological parameters, such as MNCV and sensory nerve conduction velocity (SNCV) provide important insights into the function of sciatic nerves, showing the severity of nerve injury [111]. In rodents, curcumin increased both MNCV and SNCV, showing its favorable effects on functional deficits caused by the platinum drugs [85,86]. Furthermore, curcumin attenuated alkaloid vincristine-induced sciatic functional loss by increasing level of sciatic functional index (SFI) in male Swiss albino mice [87]. The results further confirm the protective effects of curcumin against chemotherapy-induced neuropathy [87]. The improvement in histopathology of the sciatic nerve, blockade of nuclear, nucleolar atrophy, and neuronal loss supported the protective effects of curcumin against platinum-induced neurotoxicity [86,88]. Al Moundhri et al. [88] also explored co-administration of curcumin with either oxaliplatin or cisplatin and reported an insignificant reduction in the platinum concentration in the sciatic nerve. The result indicates an interesting neuroprotective activity of curcumin in which concomitant treatment of curcumin did not affect the therapeutic efficacy of platinum drugs [88]. However, more research must be conducted to further confirm the neuroprotective and anticancer activities of curcumin. Al Moundhri et al. [88] also reported that curcumin reduced oxaliplatin and cisplatin-induced increase in plasma neurotensin, providing an insight into neurotensin quantification as a biomarker of platinum-based drug neurotoxicity. Furthermore, curcumin exerted its antinociceptive activity against CIPN by modulating several markers of oxidative stress, antioxidant enzymes, and inflammatory cytokines [84,85,87]. Curcumin exerted higher efficacy in decreasing oxidative stress markers and increasing the endogenous antioxidative enzymes compared to standard drugs, including pregabalin selective Cav 2.2 (a2d subunit) channel antagonist [87]. In summary, the antinociceptive activity of curcumin against CIPN could be attributed to its multiple actions, including attenuating pain behaviors, increasing MNCV, SNCV, and SFI, and suppressing inflammatory proteins and cytokines.

### 4.3. Diabetic Painful Neuropathy (DPN)

According to a report by the Centers for Disease Control and Prevention, about 30.3 million people have diabetes, including 9.4% of adults [112]. In the United States, 50% of diabetic patients [113,114] are affected by DPN. Burning, excruciating stabbing pain, numbness, tingling sensation, paresthesia, and hyperesthesia coupled with the aching of feet or hands are some distinguished characteristic features reported in patients with DPN [115,116].

The effects of curcumin on DPN are summarized in Table 2. Curcumin received much attention in treating DPN and its associated complications in rodent models due to its relative safety and inexpensiveness [89,90,91,92,93,94,95,96,97]. Curcumin modulated STZ-induced changes in body weights in two different ways, by either increasing the STZ-induced reduction in body weight [89,95,97] or preventing/decreasing STZ-induced increase in body weight [91,93]. Hyperglycemia is the classical diagnostic marker in both type 1 and type 2 diabetes and is the major cause of diabetic neuropathy [117]. Curcumin significantly reduced elevated blood glucose levels in both mice and rat models of diabetes [93,95,97].

Pain-associated behaviors in diabetic animals are measured as exaggerated responses to painful stimuli (hyperalgesia) or nocifensive responses to normally innocuous stimuli (allodynia) [118]. Curcumin attenuated diabetes-induced thermal [89,90,91,93,95,96,97] and mechanical [89,90,91,92,93,96] hypersensitivities. Furthermore, curcumin showed protective effects against renal complications of diabetes as evidenced by a significant decrease in blood urea nitrogen (BUN), creatinine, and renal angiotensin converting enzyme 1 (ACE1) [89], an enzyme that is reported in many tissues, including kidneys and nerves [119]. The study also compared the renal protective effects of curcumin with captopril, an ACE inhibitor with antioxidant and anti-inflammatory properties [120]. However, captopril exerted significantly higher protective effects against renal complications of diabetes than curcumin did, which could be attributed to the more potent ACE inhibitory effects of captopril than curcumin.

Oxidative stress and inflammation are the two major factors that contribute to the pathophysiology of diabetes and its complications [121,122]. Curcumin ameliorated oxidative stress in DPN models by increasing the key enzymes for antioxidant defense, such as SOD [92], and by increasing total antioxidant capacity (TAS) [94]. TAS provides protection from the neurological damage caused by diabetes-induced oxidative stress [123,124]. It also provides important information on the total antioxidant content in a biological system [123,124]. Curcumin also exerted its antioxidant properties by reducing or scavenging several oxidative stress markers, such as the lipid peroxidation marker MDA [92,94,96], hydrogen peroxide (H_2_O_2_) [92], nitric oxide (NO) [94,95,96,97], and oxidative stress index (OSI) and total oxidant status (TOS) [94]. OSI and TOS indicate a total concentration of all free radicals generated by diabetes-related oxidative damage [123,125]. Zhao et al. [92] reported that curcumin ameliorated the protein expressions of nicotinamide adenine dinucleotide phosphate reduced form (NADPH) oxidase subunits gp91^phox^ and p47^phox^. The phosphorylation activates NADPH oxidases, leading to the generation of ROS, including H_2_O_2_. Therefore, decreases in gp91phox and p47phox could lead to the decrease in oxidative stress [126,127,128,129]. In addition, curcumin exerted its anti-inflammatory properties by decreasing the production of pro-inflammatory cytokine TNF-α [89,93,95,96,97] or its receptor 1 (TNF-α receptor 1) [93]. In comparison with a standard antioxidant apocynin, curcumin demonstrated similar antioxidant activity against DPN [92] (Table 2).

### 4.4. Sciatic Nerve Chronic Constriction Injury (CCI)

Animal models of CCI are widely used to study peripheral neuropathic pain. The CCI model of nerve injury possesses two components, inflammatory and nerve injuries, which resemble the pain found in humans [130].

Table 3 summarizes the effects of curcumin on sciatic nerve CCI. Similar to other neuropathic pain models, curcumin alleviated CCI-induced neuropathic pain behaviors, including heat [131,132,133,134,135], mechanical [92,131,132,133,135,136], and cold [137] hypersensitivities. However, Moini Zanjani et al. [137] reported that low doses (12.5 and 25 mg/kg) of curcumin did not reduce pain behavior but induced mechanical allodynia. However, a high dose (50 mg/kg) of curcumin reduced cold allodynia [137]. The results indicate that different doses of curcumin are effective in alleviating CCI-induced pain behaviors. Curcumin alleviated CCI-induced neuropathic pain by inhibiting the expression of nuclear factor kappa B (NF-κB) in the spinal cord and reducing the expression of CX3C chemokine receptor 1 (CX3CR1) in the dorsal spinal cord and DRG [131] as well as attenuating the messenger RNA (mRNA) or protein expressions [135] of an important inflammatory mediator, cyclooxygenase-2 (Cox-2) [138], and its serum level [137]. Cox-2 is constitutively expressed in the dorsal horn of the spinal cord and is upregulated following injury, leading to the transmission of nociceptive input [139,140]. Besides modulating Cox2, curcumin also reduces the serum level of cortisol by inhibiting the upregulated expression of 11β-hydroxysteroid dehydrogenase type I enzyme (11βHSD1) [132]. The 11βHSD1 is a key enzyme that converts cortisone to cortisol in humans, and 11-dehydrocorticosterone to corticosterone in rodents [141]. These glucocorticoids exert their effects through glucocorticoid receptors that play important roles in the maintenance and development of neuropathic pain by regulating the function and expression of N-methyl-D-aspartate receptor (NMDAR) [142]. Yu et al. [133] reported that curcumin exerted anti-allodynic activity by blocking the immunohistochemical and protein expressions of N-methyl-D-aspartate receptor subunit NR1 (NMDAR NR1) in the spinal cord and DRG. However, Jeon et al. [136] reported that curcumin did not change the protein expression of NR1 in DRG. This discrepancy could be attributed to the dose and duration of curcumin treatment. Yu et al. employed 100 mg/kg of curcumin for 14 days [133], whereas Jeon et al. [136] used 50 mg/kg of curcumin for 7 days in rats. The treatment for 7 days [136] was probably too short to see the curcumin-induced changes at the central sensitization. Therefore, future studies are required to examine the long-term treatment of curcumin on central sensitization in rodent chronic neuropathic pain model. In addition, NMDAR-mediated activation of brain-derived neurotrophic factor (BDNF) is associated with the enrichment of p300/CREB-binding protein (CBP) at the BDNF gene promoter I [143]. Curcumin exerted its therapeutic activity by downregulating the recruitment of p300/CBP and histone acetyltransferase (HAT) (acetyl-Histone H3/acetyl-Histone H4) to the BDNF promoter [135]. Curcumin also downregulated p300/CBP HAT activity-mediated gene expression of Cox-2 [135].

Curcumin turned out to be less efficacious in a chronic constriction injury-chronic constriction release (CCI-CCR) model of neuropathic pain when compared to a neuropathic drug tramadol hydrochloride, a synthetic opioid from the aminocyclohexanol group [145]. However, curcumin was effective in inducing high regeneration and decreasing degeneration of nerve tissues in CCR compared to tramadol [144]. Findings from Ceyhan et al. [144] indicate that long-term use of curcumin in surgical constriction release may exert beneficial effects in ameliorating CCI-induced neuropathic pain.

Zhao et al. [134] explored the underlying mechanisms of antinociceptive action of curcumin in CCI-induced neuropathic pain. The study proposed that descending monoamine system spinal beta2-ARs and delta opioid receptors maintain the anti-allodynic activity of curcumin on mechanical stimuli, whereas descending serotonergic system coupled with spinal 5-HT1A receptors and mu opioid receptors are required for the anti-hyperalgesic activity of curcumin on thermal stimuli [134].

### 4.5. Other Peripheral Neuropathic Pain Models

#### 4.5.1. Sciatic Nerve Crush (SNC) Injury

Compression, fracture, crush, wound, and laceration lead to the injury of sciatic nerves [146,147], and to the partial or total autonomic, motor, and sensory function loss [148]. A sciatic nerve crush model in rodents is widely used to represent axonotmesis-like moderate peripheral neuropathy (PN) injury and is characterized by myelin sheath destruction and Wallerian degeneration [149].

Table 4 summarizes the effects of curcumin on SNC. Curcumin demonstrated neuroprotective effects on peripheral nerve injury by promoting nerve regeneration [150,151,152] and protecting the injured DRG and sciatic nerve structures [153,154]. In a combination study, curcumin was administered with melatonin, a drug that is used in nerve tissue recovery and repair [155]. Since melatonin is affected by light and dark, the study comparatively evaluated the effects of curcumin and melatonin in light and dark periods [156]. The results showed that curcumin exerted better efficacy in stimulating nerve regeneration compared to melatonin. However, the effects of curcumin did not differ between the light and dark periods of treatments, but melatonin showed significantly better efficacy in the dark compared to light group [151]. Therefore, future studies should explore the effects of curcumin in human nerve regeneration. Furthermore, Ma et al. [152] reported that high doses of curcumin (100 mg/kg and 300 mg/kg) induced similar nerve regeneration effects as mecobalamin, a neuroprotective agent commonly used as a neuroprotective agent against neurodegenerative diseases [157]. All the evidence reinforces the neuroprotective effects of curcumin in promoting nerve regeneration and accelerating motor functional recovery.

#### 4.5.2. Spared Nerve Injury (SNI)

SNI model resembles the stimulus-evoked pain that is observed under clinical settings of neuropathic pain syndrome [162,163]. Table 4 summarizes the effects of curcumin on SNI. Curcumin reduced SNI-induced neuropathic pain behaviors by activating either the tropomyosin receptor kinase A (TrkA) and phosphatidylinositol 3-kinase/Akt protein kinase B (PI3K/Akt) cell survival signaling pathway [158] or the Janus kinase 2-signal transducer and activator of transcription 3 (JAK2-STAT3) signaling pathway [159]. Nerve damage induces neuroinflammation [164], which leads to the upregulation of pro-inflammatory cytokines [164], including IL-1β, that contribute to the development and maintenance of neuropathic pain [165]. Curcumin downregulated the production of mature IL-1β in the spinal cord and thus attenuated SNI-induced neuropathic pain [159]. Furthermore, curcumin induced the anti-allodynic activity by inhibiting the NAcht leucine-rich-repeat protein 1 (NALP1) inflammasome and activating the JAK2-STAT3 pathway in astrocytes [159]. On the other hand, curcumin demonstrated protective effects against injured neurons by stimulating the release of nerve growth factor (NGF) and further activating the TrkA and PI3K/Akt cell survival signaling pathway [158].

#### 4.5.3. Spinal Nerve Ligation (SNL)

Kiso et al. [166] developed a L5/L6 mice spinal nerve ligation model, which is employed in studying neuropathic pain. In this nerve ligation model, mechanical allodynia develops at day 1 and lasts for two months after the surgery.

Table 4 summarizes the effects of curcumin on SNL. Lee et al. [161] reported that intrathecal administration of curcumin alleviated SNL-induced allodynia, but they did not explore the underlying mechanisms of action. On the other hand, Pastrana-Quintos et al. [160] reported that both oral and intrathecal curcumin induced anti-allodynic activity in an SNL model of neuropathic pain and that the anti-allodynic effect was mediated via the nitric oxide-cyclic guanosine monophosphate-adenosine triphosphate-sensitive potassium + channels pathway. Furthermore, the highest dose of oral (310 mg/kg) and intrathecal (0.3 mg) curcumin exerted maximal anti-allodynic effects, and intrathecal curcumin even produced significantly higher anti-allodynic activity compared to gabapentin [160].

## 5. Curcumin and Postoperative Pain and Preemptive Analgesia—Preclinical Studies

### 5.1. Postoperative Pain

Patients perceive postoperative pain as one of the most noxious aspects of surgical pain for which effective control measures are lacking [167,168,169]. Table 5 summarizes the effects of curcumin on postoperative pain and preemptive analgesia. Acute treatment of curcumin demonstrated anti-hyperalgesic activity by dose-dependently reversing mechanical hyperalgesia, whereas repeated treatment facilitated the recovery of postoperative pain [170]. However, repeated treatment before surgery did not exert impact on the prevention or reduction in postoperative pain [170]. The results emphasize that acute curcumin treatment may be useful in treating postoperative pain. Curcumin also exerted its analgesic activity by alleviating incision-induced inflammation, spontaneous pain, functional gait abnormalities, and hyperalgesic priming [171]. Although curcumin did not alter the pro- or anti-inflammatory cytokines at the peri incisional level, it augmented transforming growth factor-β (TGF-β), which is implicated to inhibit nociception in both inflammatory and neuropathic pain models [172]. Ju et al. [173] provided important insights into the underlying mechanisms of the antinociceptive activity of curcumin in postoperative pain. The results showed that antagonizing the gamma-aminobutyric acid (GABA) receptors abrogated the curcumin-induced anti-hyperalgesic activity, and curcumin treatment elevated the mRNA expression of GABA-A and GABA-B in the incised spinal cord. On the other hand, antagonizing the opioid receptors reversed the anti-hyperalgesic activity of curcumin but did not alter the mRNA expression of opioid receptors in the spinal cord, indicating the indirect involvement of opioid receptors in mediating curcumin antinociception of postoperative pain [173]. Together, the findings conclude that spinal GABA receptors are important in modulating postoperative pain and that curcumin increases the synthesis of GABA mRNA in the spinal cord, thus mediating the antinociception of postoperative pain. Therefore, postoperative pain can be treated or prevented with spinal GABA receptor agonists.

### 5.2. Preemptive Analgesia

Preemptive analgesia is an antinociceptive treatment that is applied to prevent altered processing of the afferent input that amplifies postoperative pain by preventing central sensitization caused by incisional and inflammatory injuries, and it covers both the period of surgery and the initial postoperative period. The nature of surgery determines the balance between incisional injury and inflammatory injury, with inflammation injury being a dominant factor [177,178]. The application of preemptive analgesia is more effective in reducing surgery-induced nociceptive pain transmission when compared to the application of analgesic treatment provided after surgery [179].

Nurullahoglu et al. [176] suggested the preemptive analgesic effects of curcumin on acute thermal and inflammation-induced pain in female Wistar Albino rats. Furthermore, the study compared the preemptive effects of curcumin with intraperitoneal administration of diclofenac (10 mg/kg), a non-steroidal anti-inflammatory drug [180]. Diclofenac exerts controversial preemptive effects, with some studies, showing no differences in the effects in between pre- and postoperative diclofenac-treated patients undergoing laparoscopic tubal ligation [177], while other studies reported that preoperative administration of diclofenac along with ketorolac and piroxicam reduced postoperative pain in patients undergoing laparoscopy [181,182]. Based on Nurullahoglu et al.’s [176] study, both curcumin and diclofenac exerted preemptive analgesic effects. Bulboacs et al. [175] demonstrated the preemptive effects of curcumin in a rodent migraine model. Curcumin induced analgesic effects in both phase I dominated by vasodilation and phase II dominated by inflammation of formalin test [175]. Moreover, curcumin reduced oxidative stress markers and blood pressure and increased TAC. The study also compared the preemptive analgesic effects of curcumin with a beta-1 blocker, propranolol [183], which is effective in treating migraine patients by increasing the temporal distances between migraine attacks [184]. Another drug, indomethacin, exerts antimigraine effects due to its antinociceptive and anti-inflammatory properties [185]. Bulboacs et al. [175] demonstrated that curcumin had superior activity as compared to propranolol- and indomethacin-treated groups, indicating that curcumin could be used as prophylaxis for migraine. In addition to rodent models, the preemptive analgesic property of curcumin was also effective in a swine model of cardiopulmonary bypass (CPB) and extracorporeal support, resulting in a decrease in TNF-α and intercellular adhesion molecule (ICAM-1) expressions [174]. This study in a swine model provides data for the development of a human translational study [174]. However, further studies are needed to explore the underlying mechanisms of preemptive analgesic effects of curcumin.

## 6. Curcumin Formulations and Neuropathic Pain—Preclinical Studies

Preclinical and clinical studies have employed different curcumin formulations synthesized in order to improve the solubility, bioavailability, and pharmacokinetics of curcumin [186,187,188,189,190].For example, in a clinical study, a novel bio-enhanced preparation of curcumin called BCM-95CG (Biocurcumax) showed 6.93- and 6.3-fold higher bioavailability when compared to curcumin and a curcumin-lecithin-piperine formula, respectively [186]. However, Shoba et al. [189] reported that concomitant administration of piperine enhanced the bioavailability, absorption, and serum concentration of curcumin in both rodents and humans with no side effects. Another curcumin formula, Theracurmin, which is curcumin dispersed with colloidal submicron particles, exhibited higher absorption efficiency compared to other curcumin drug-delivery systems, such as BCM-95 (micronized curcumin with turmeric essential oils) and Meriva (curcumin-phospholipid) [187]. A curcumin formulation with a combination of hydrophilic carrier, cellulosic derivatives, and natural antioxidants further showed higher absorption in blood compared to unformulated curcumin [188].

**Figure 3:**
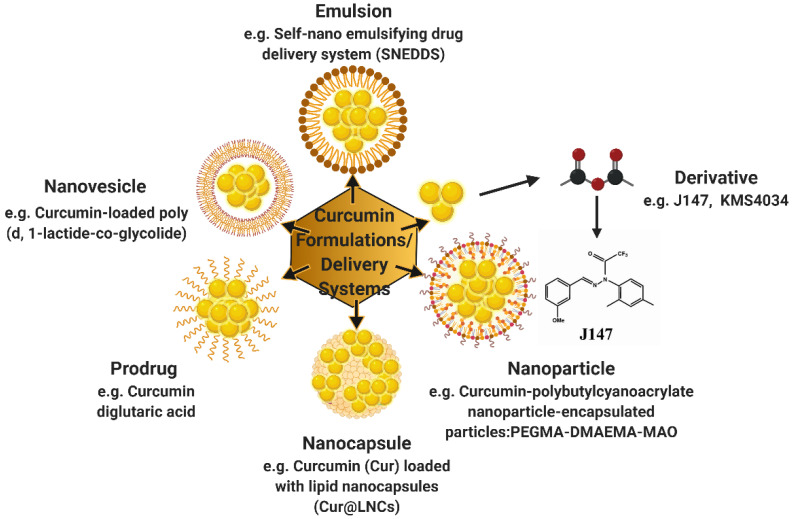
Schematic representations of different curcumin formulations and delivery systems along with their respective examples discussed in the review (created with BioRender).

However, only a few studies have looked into the effects of curcumin formulations in neuropathic pain, specifically diabetic and CCI-induced neuropathies [27,28,29,30,31,32,33,34] (Figure 3). Table 6 summarizes the effects of different curcumin formulations on neuropathic pain. Curcumin derivative J147 was reported to possess potent neurogenic and neuroprotective activities and was initially developed to treat neurodegenerative conditions [191,192]. J147 exerted its therapeutic potential by reducing multiple pathogenic pathways associated with the DPN in rodent models [27,33]. Reduced AMP kinase signaling is known to be associated with DPN [193,194]. J147 stimulated the AMPK signaling pathway by increasing its protein and mRNA expressions [27,33]. J147 also ameliorated diabetes-induced mechanical hypersensitivity [27,33], heat hypersensitivity [27], and increased MNCV [27]. Curcumin delivery systems, such as the self-nano emulsifying drug delivery system (SNEDDS) [30] and nanoparticle-encapsulated curcumin (curcumin-polybutylcyanoacrylate nanoparticle-encapsulated particles: PEGMA-DMAEMA-MAO) [28] improved diabetes-induced mechanical and thermal hypersensitivities [28,30]. The nanoparticle-encapsulated curcumin decreased the mRNA and protein expressions of purinergic receptor 12 (P2Y12), which is expressed on satellite glial cells (SGCs) in DRG [195,196]. P2Y12 is activated by ATP and ADP and plays an important role in transmitting painful signaling [195,197,198,199]. The decreased expression of P2Y12 by nanoparticle-encapsulated curcumin led to the decrease in pro-inflammatory cytokines IL-1β and connexin 43 (Cx43) and ultimately decreased neuronal excitability in the DRG and resulted in attenuation of diabetes-induced painful behavior [28]. Furthermore, SNEDDS more efficiently reversed diabetes-induced functional, sensorimotor, and biochemical deficits by decreasing neuroinflammation and improving the antioxidative defense system as compared to naïve curcumin [30]. Besides diabetes, curcumin formulae are also effective in attenuating CCI-induced neuropathic pain by decreasing pronociceptive peptides [161] or pro-inflammatory cytokines [32]. A curcumin formula, curcumin-loaded poly (d, l-lactide-co-glycolide) nanovesicles (PLGA-CUR), at both low and high doses, attenuated CCI-induced pain behaviors [34]. The results further confirm the better efficacy of curcumin formulations over parent curcumin, which possesses poor bioavailability and is required at high doses to attenuate neuropathic pain.

Together, the efficacy of these curcumin formulations against neuropathic pain supports their applications in clinical investigation. Eventually, further improvement of curcumin formulations would enhance their use as adjuvants to neuropathic drugs.

## 7. Curcumin and Its Formulations on Neuropathic Pain or Postoperative Pain—Clinical Studies

A vast majority of the studies have reported the antioxidant and anti-inflammatory properties of curcumin and its formulations in clinical settings of chronic inflammatory joint pain, such as osteoarthritis and rheumatoid arthritis [200,201,202,203,204,205,206,207,208]. Only a few clinical studies have focused on the effects of curcumin and/or its formulations in PN and postoperative pain [209,210,211,212,213,214,215].

Table 7 summarizes the effects of curcumin on neuropathic pain and postoperative pain in clinical studies. Diabetic sensorimotor polyneuropathy (DSPN) is one of the most common complications in diabetes mellitus, resulting in impaired motor activity [216]. DSPN affects 25% of individuals with type 2 diabetes mellitus (T2DM) [217,218]. Asadi et al. [211] reported that nano curcumin supplementation decreased the total neuropathy score when assessed by the Toronto Clinical Neuropathy Score. The nano curcumin treatment also reduced the serum levels of fasting blood glucose (FBS) and HbA1c. The study also elucidated that DSPN can be improved by managing hyperglycemia in individuals with T2DM. In another study, patients treated with Meriva (lecithinized curcumin) showed significantly reduced chemotherapy-induced side effects, which was further confirmed by the semiquantitative evaluation of chemotherapy-induced side effects in the control group. Furthermore, patients treated with Meriva had reduced plasma levels of free radicals when compared to the control group [212]. Curcumin is also effective against chronic PN and pain induced by lumbar disc herniation and/or lumbar canal stenosis or carpal tunnel syndrome [215]. A multi-ingredient formula (800 mg dexibuprofen (Dex) + Lipicur (800 mg lipoic acid + 800 mg curcumin phytosome + 8 mg piperine), Dex + 800 mg lipoic acid, and 800 mg Dex only) reduced neuropathic pain in patients with lumbar sciatica and carpal tunnel syndrome. Curcumin efficiently reduced the use of dexibuprofen by 40%, and add-on therapy with lipoic acid exerted no significant results, indicating that Lipicur could be used as an effective alternative therapeutic to treat neuropathic pain [215].

In a pilot randomized trial, curcuminoids extracted from turmeric, containing curcumin, demethoxycurcumin, and bisdemethoxycurcumin [219], reduced postoperative pain severity after laparoscopic gynecologic surgery [214]. In another double-blinded, randomized, placebo-controlled study, curcumin not only reduced postoperative pain but also reduced fatigue based on the patient-reported outcomes following laparoscopic cholecystectomy [209]. Curcumin also reduced intensity of the acute postoperative pain followed by third molar extraction as evaluated by numeric rating scale [213]. Curcumin exerted better efficacy in reducing orofacial pain caused by the postoperative molar extraction-induced inflammation in comparison with mefenamic acid, a non-steroidal anti-inflammatory drug, commonly used to treat inflammatory pain [220,221,222]. Postsurgical removal of the third molar led to the upregulation of inflammatory cytokines, including interleukin 6 (IL-6) and interleukin 8, leading to the development of inflammatory pain [223]. Therefore, in their study, Maulina et al. [213] explained that curcumin demonstrated better anti-inflammatory activity by directly inhibiting the inflammatory cytokines as compared to mefenamic acid that indirectly inhibited or decreased IL-6 by suppressing the secretion of prostaglandin E2, responsible for inducing IL-6 expression [224]. The analgesic activity of curcumin against periodontal surgeries was further confirmed in another study in which curcumin mucoadhesive film reduced postoperative pain and swelling over a period of one week compared to the placebo mucoadhesive film. Therefore, the curcumin mucoadhesive film could become a commercially available phytochemical drug delivery system in the treatment of periodontal postsurgical pain [210]. Taken together, these aforementioned clinical studies conclude that curcumin and its formulations could be used as adjuvants for postoperative care.

## 8. Conclusions

**Figure 4:**
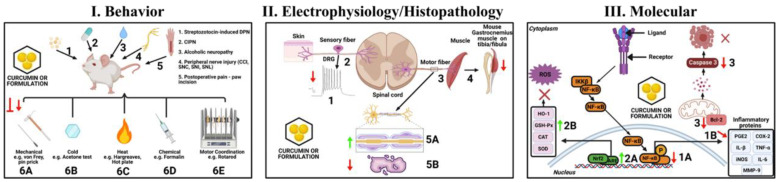
Effects on curcumin or its formulations at the behavior, electrophysiology/histopathology, and molecular levels in peripheral neuropathic and postoperative pain. **I. Behavior:** Curcumin or its formulations inhibit or reduce DPN (1), CIPN (2), alcoholic neuropathy (3), different peripheral injuries, such as CCI, SNC, SNI, SNL, etc., and/or postoperative pain-induced behaviors in rodent models. Curcumin and its formulations mainly inhibit or reduce mechanical (6A), cold (6B), heat (6C), and chemical-induced (6D) pain behaviors, as well as motor deficits (6E). **II. Electrophysiology/Histopathology:** Curcumin or its formulations protect injured DRG, decrease neuronal excitability in DRG (1), resulting in attenuation of painful neuropathic behavior. Curcumin or its formulations increase SNCV, decrease loss of DRG neurons, and increase diameter of nerve fibers (2). Furthermore, curcumin and its formulations increase MNCV, decrease neurogenic lesions (3), and atrophy of gastrocnemius muscle (4). The treatments also effectively increase myelin sheath thickness (5A) and prevent demyelination (5B). **III. Molecular:** Curcumin or its formulations decrease expression of NF-κB, leading to decrease in inflammatory proteins. Furthermore, the treatments increase expressions of Nrf2, leading to increase in levels of antioxidative enzymes that scavenge free radicals and ultimately reduce ROS levels. Moreover, the treatments decrease expressions of Bcl-2 and caspase-3 that lead to reduction in apoptosis and ultimately improve nerve injuries (created with BioRender).

The current review provides important information regarding the potential effects of curcumin in treating different peripheral neuropathic conditions, including alcoholic neuropathy, CCI-, CIPN-, DPN-, SNI-, and SNL-induced neuropathic and postoperative pain (Figure 4). Based on the present review, we identified a few drawbacks in both preclinical and clinical studies. First, only a handful studies have explored the effects of curcumin and its formulations in neuropathic and postoperative pain under clinical settings. Future studies should focus on conducting clinical studies on other PN pain conditions involving curcumin. Second, in most of the clinical studies only a single dose was used. Therefore, dose-related effects remain unknown. Third, only a few preclinical studies have compared the antinociceptive effects of curcumin with standard drugs. In order to enhance the application of curcumin in clinical treatment, it is important to administer clinically used drugs as experimental controls or for a reference comparison. Fourth, most of the animal studies evaluated the anti-hyperalgesic activity of curcumin either by monitoring the behavioral outcomes or by measuring the biochemical paraments. Besides these two parameters, the effects of curcumin on neuropathic pain should also be evaluated by monitoring functional recovery and electrophysiological aspects of pain conditions. Therefore, in the future, it is important to address the aforementioned shortcomings while designing both preclinical and clinical studies. In conclusion, with the advent of these new formulations, including curcuminoids, liposomal encapsulations, nanoparticles, derivatives, and analogs, the multifaceted favorable effects of curcumin will lead to the promising development of therapeutic agents for treating several neuropathic and postoperative pain conditions.

## Figures and Tables

**Table 1:** Serum and tissue levels of curcumin in animal and clinical studies.

Animals	Dose ^a^, Route of Administration	Bioavailability			Reference
		Tissue	Concentration	Time Measured after Administration	
**Animal Studies**					
NMRI and C57/BL6 mouse	50 mg/kg, force-fed	Brain	Below detection limit	30, 60, and 120 min	
	100 mg/kg, i.p.		0.004–0.005 mg/g	20–40 min	[65]
C57BL6/J male and female mice	0.148 mg, i.p.	Brain	0.000739 ± 0.019 mg/g 2.010 µM	4 h	[55]
		Plasma	0.000127 ± 0.035 mg/mL 0.345 µM		
	0.148 mg, oral	Brain	0.000519 ± 0.098a mg/g 1.412 µM	4 h	
		Plasma	Below detection level		
	0.074 mg, intramuscular	Brain	0.001162 ± 0.004 mg/g 3.157 µM	4 h	
		Plasma	0.000238 ± 0.048 mg/mL 0.647 µM		
	~2.5 mg/day, oral (500 ppm)	Brain	0.000469 ± 0.220 mg/g 1.276 µM	4 months	
		Plasma	0.000035 ± 0.014 mg/mL 0.095 µM		
	~10 mg/day, oral (2000 ppm)	Brain	0.000525 ± 0.125 mg/g 1.428 µM	4 months	
		Plasma	0.000171 ± 0.019 mg/mL 0.465 µM		
Male Sprague-Dawley rats	500 mg/kg, oral	Plasma	0.00006 ± 0.01 mg/mL	41.7 ± 5.4 min	[56]
	10 mg/kg, intravenous	Plasma	0.00036 ± 0.05 mg/mL	Not mentioned	
Male Sprague-Dawley rats	1000 mg/kg, oral	Plasma	15 ng/mL	50 min	[66]
Female BALB/c mice	0.1 g/kg, i.p.	Brain	0.00041 ± 0.01 mg/g	1 h	[50]
		Intestine	0.11704 ± 6.86 mg/g		
		Kidneys	0.00751 ± 0.08 mg/g		
		Liver	0.0269 ± 2.58 mg/g		
		Plasma	0.0006 ± 0.03 mg/g		
		Spleen	0.02606 ± 1.06 mg/g		
**Clinical Studies**					
Human	500–8000 mg/day, oral	Serum	0.51 ± 0.11 µM 0.63 ± 0.06 µM 1.77 ± 1.87 µM	1–2 h	[67]
	4000 mg 6000 mg 8000 mg	Urine	Undetectable		
Human	500–12,000 mg, oral 500–8000 mg	Serum	Undetectable	1, 2, 4 h	[63]
	10,000 mg		30.4 ng/mL 39.5 ng/mL 50.5 ng/mL	1 h 2 h 4 h	
	12,000 mg		29.7 ng/mL 57.6 ng/mL 51.2 ng/mL	1 h 2 h 4 h	
Human	0.45–3.6 g, oral	Plasma	11.1 ± 0.6 nmol/L	1 h	[68]
	Under 3.6 g	Urine	0.1–1.3 nmol/L		
Human	2 or 4 g, oral	Plasma	7 ng/mL	24 weeks	[69]
Human	100 mg, oral	Plasma	3.2 nM	2 h	[70]
Human	8 g, oral	Plasma	29 to 91 ng/mL	3 months	[71]

^a^ = Varies based on the studies mentioned in the table; i.p. = intraperitoneal.

**Table 2:** Effects of curcumin on alcoholic neuropathy, chemotherapy-induced peripheral neuropathy (CIPN), and diabetic painful neuropathy (DPN).

Animals (Sex, Strain)	Dose (mg/kg), Route of Administration, Duration of Treatment	Effects			Reference
		Behavioral/Other	Electrophysiological/ Functional	Histopathological/ Biochemical/Molecular	
**Alcoholic Neuropathy**					
Male Wistar rats	35% (*v*/*v*) ethanol 10 g/kg, b.i.d (bis in die, i.e., twice daily), oral, 10 weeks + curcumin: 20, 40 and 80 mg/kg, oral, 10 weeks + 35% (*v*/*v*) ethanol (10 g/kg), oral, 10 weeks	↑ Mechanical hyperalgesia threshold (Randall–Selitto paw pressure test) ↓ Mechanical allodynia (von Frey hair test) Thermal hyperalgesia (Tail immersion test)	**X** Reduction in MNCV	↓ MDA, neural nitrite, and total calcium content ↓ TNF-α and IL-1β and DNA fragmentation in sciatic nerve	[80]
**Combination Study**					
Wistar Albino rats of either sex	Curcumin per se: 60 mg/kg, i.p. 10 weeks 35% (*v*/*v*) ethanol (10 g/kg, twice daily, oral, 10 weeks) + curcumin (30 and 60 mg/kg, oral, 10 weeks) Sildenafil per se: 10 mg/kg, i.p. 10 weeks 35% (*v*/*v*) ethanol (10 g/kg, twice daily, oral, 10 weeks) + sildenafil (5 and 10 mg/kg, oral, 10 weeks) 35% (*v*/*v*) ethanol (10 g/kg, twice daily, oral, 10 weeks) + curcumin (30 mg/kg, oral, 10 weeks) + sildenafil (5 mg/kg, oral, 10 weeks)	Combination **✓** Improved motor coordination (rotarod test) ↓ Thermal hyperalgesia (hotplate test) and paw heat allodynia (hotplate test), mechanical hyperalgesia (pin prick test), cold allodynia (acetone test), tail cold-hyperalgesia (tail immersion test)	Not tested	↓ MDA level **✓** GSH level ↓ Ethanol-induced fiber derangement, nerve fiber swelling, Schwann cells activation **✓** Nerve fibers	[83]
**Chemotherapy-Induced Peripheral Neuropathy (CIPN)**					
Male Sprague-Dawley rats	Paclitaxel (2 mg/kg, i.p., 5 consecutive days) Curcumin (200 mg/kg/day, oral, 10 consecutive days) Paclitaxel (2 mg/kg, i.p., 5 consecutive days) + curcumin (100 or 200 mg/kg/day, oral, 10 consecutive days)	Not tested	Not tested	In spinal cord and sciatic nerve tissues: ↓ NF-κB and GFAP levels ↓ mRNA expressions of TNF-α, IL6, NF-κB, iNOS, Bcl-2 and Bcl-xL ↑ mRNA expression of Nrf2 ↓ mRNA expressions of caspase-3, p53, Apaf-1 ↓ mRNA expressions of autophagy related genes LC3A, LC3B, and Beclin-1 ↓ Immunohistochemical expressions of 8-OHdG, caspase-3 and LC3B **✓** Spinal cord and sciatic nerve histological architecture and integrity with no sciatic nerve damage, including vacuolation, neuronal degeneration, and demyelination	[84]
Male Sprague-Dawley rats	Oxaliplatin (4 mg/kg, i.p., twice weekly for 4 weeks) + curcumin (12.5, 25 and 50 mg/kg, oral, 4 weeks)	↑ Mechanical (von Frey) threshold ↓ Cold allodynia (acetone test)	↑ MNCV and SNCV	**✓** Injured spinal cord cells ↑ Levels of SOD, GSH-Px, and CAT in spinal cord ↓ MDA level in spinal cord **X** Protein expression of p-NF-κB/NF-κB in spinal cord ↓ TNF-α, IL-6 and IL-1β levels in spinal cord	[85]
Female Wistar rats	Cisplatin (2 mg/kg, i.p., twice a week for 5 weeks) + curcumin (200 mg/kg, oral, once daily for 5 weeks)	↓ Thermal hypoalgesia (hotplate test)	**✓** Reduction in MNCV	↑ Myelin thickness in sciatic nerve **X** Nuclear and nucleolar atrophy, loss of neurons in L4 DRG	[86]
Male Swiss Albino mice	Vincristine sulfate (0.1 mg/kg, i.p., once per day for 7 consecutive days) + curcumin (15, 30, 60 mg/kg/day, oral, 14 consecutive days)	↑ Thermal hyperalgesia (hotplate test) ↓ Thermal allodynia (cold plate test) and mechanical hyperalgesia (pin prick test) **O** Motor coordination (rotarod test) **O** Acute phase of nociception (formalin test) ↓ Paw elevation and licking in delayed phase of nociception (formalin test)	↓ Vincristine-induced rise in SFI	↓ Calcium level in sciatic nerve ↑ SOD activity in sciatic nerve ↑ Sciatic SOD, CAT, GPx, and GSH ↓ Levels of LPO and NO	[87]
Male Wistar rats	Oxaliplatin (4 mg/kg, i.p., twice weekly for 4.5 weeks) + curcumin (10 mg/kg, oral, twice weekly for 4.5 weeks) Cisplatin (2 mg/kg, i.p., twice weekly for 4.5 weeks) + curcumin (10 mg/kg, oral, twice weekly for 4.5 weeks)	**O** Motor coordination (rotarod test), cold (cold water tail flick test), mechanical (paw pressure test), thermal (tail flick test) nociception	Not tested	↓ Plasma concentration of neurotensin Co-treatment with oxaliplatin or cisplatin insignificantly ↓ platinum concentration in sciatic nerve ↓ Demyelination	[88]
**Diabetic Painful Neuropathy (DPN)**					
Male Wistar rats	Streptozotocin (STZ) (100 mg/kg, i.p.) + curcumin (100 mg/kg, oral, 6 weeks)	↑ Body weight, and kidney weight/body weight ↑ Thermal hyperalgesia (hotplate and tail flick test) and mechanical allodynia (von Frey)		↓ FBG, TG, total cholesterol, LDL-C, total peroxide, serum creatinine, and BUN ↑ HDL-C ↓ Renal ACE1 **✓** TNF-α and IL-10 in kidneys and sciatic nerves	[89]
Male Sprague-Dawley rats	^a^ Animals with type 2 diabetes with diabetic neuropathic pain + curcumin (100 mg/kg, i.p., 14 days)	↑ Mechanical withdrawal threshold (von Frey) and thermal withdrawal latency (heat stimulus)	**✓** TTX-R sodium currents I_Na_ of small-sized DRG neurons	Not tested	[90]
Male Albino Wistar rats	STZ (60 mg/kg, i.p.) + acute or chronic curcumin (50 mg/kg/day, i.p.) Acute treatment: only 30 min prior to pain assessment Chronic treatment: from 7th day till 21st day injected once a day	**O** Hyperglycemia Chronic treatment **X** Weight loss Chronic treatment ↑ Thermal hyperalgesia (plantar test) and mechanical allodynia (von Frey) Naloxone pre-treatment ↓ Anti-allodynic effect of chronic curcumin	Not tested	Not tested	[91]
Male Sprague-Dawley rats	STZ (60 mg/kg, i.p.) + curcumin (200 mg/kg, intragastric, 14 days)	**O** Hyperglycemia and increased body weight ↑ Tactile allodynia (von Frey)	Not tested	**✓** Protein expressions of NADPH oxidase subunits gp91^phox^ and p47^phox^ in spinal cord ↓ H_2_O_2_ and MDA in spinal cord ↑ SOD in spinal cord	[92]
Male Sprague-Dawley rats	STZ (65 mg/kg, i.p.) + curcumin (60 mg/kg, oral, daily from day 3 to day 28)	↓ Hyperglycemia and body weight **✓** Thermal hyperalgesia (Hargreaves test) and mechanical allodynia (von Frey)	Not tested	↓ Spinal TNF-α and TNF-α receptor 1	[93]
Female Wistar Albino rats	STZ (50 mg/kg, i.p.) + curcumin (60 mg/kg, oral, 21 days) Non-diabetic rats + curcumin (60 mg/kg, oral, 21 days)	Not tested	Not tested	↓ MDA, TOS, OSI, and NO in brain and sciatic tissues ↑ TAS in brain and sciatic tissues	[94]
Male Albino mice of Laka strain	STZ (200 mg/kg, i.p.) + curcumin (15, 30, and 60 mg/kg, oral,4th 8th week)	↓ Plasma glucose level ↑ Body weight ↑ Thermal hyperalgesia (tail immersion warm water test and hotplate test)	Not tested	↓ Serum TNF-α level ↓ Brain nitrite level	[95]
**Combination Studies**					
Male Sprague-Dawley rats	STZ (45 mg/kg, i.p.) + gliclazide (10 mg/kg, oral, 5 weeks) STZ (45 mg/kg, i.p.) + gliclazide (10 mg/kg, oral, 5 weeks) + gabapentin (30 mg/kg, i.p., 5 weeks) STZ (45 mg/kg, i.p.) + gliclazide (10 mg/kg, oral, 5 weeks) + curcumin (100 mg/kg, oral, 5 weeks)	↑ Thermal hyperalgesia (hotplate and tail flick test), and mechanical hyperalgesia (tail pinch test)	Not tested	****✓**** C-peptide level ↓ Total NO, serum TNF-α and MDA	[96]
Male Albino mice of Laka strain	STZ (200 mg/kg, i.p.) + insulin (10 IU/kg, s.c., 8 weeks) STZ (200 mg/kg, i.p.) + curcumin (60 mg/kg, oral, 8th weeks) STZ (200 mg/kg, i.p.) + resveratrol (20 mg/kg, oral, 8th weeks) Insulin (10 IU/ kg, s.c., 8 weeks) + curcumin (60 mg/kg, oral, 8th weeks) Insulin (10 IU/ kg, s.c., 8 weeks) + resveratrol (20 mg/kg, oral, 8th weeks)	Insulin per se ↓ Blood glucose level and ↑ body weight Curcumin or resveratrol per se ↓ Blood glucose level and ↑ body weight Combination treatment **O** blood glucose level and body weight Combination treatment ↑ thermal hyperalgesia (tail immersion warm water test and hotplate test) threshold	Not tested	Combination treatment ↓ Serum TNF-α level compared to its per se effects Combination treatment ↓ Brain nitrite level compared to its per se effects	[97]

Behavioral modalities are mentioned within parentheses. ^a^ = Induction of type 2 diabetes with diabetic neuropathic pain: one group of animals fed high fat–fructose diet (HFD) for eight weeks and another group received normal feeding. After significant differences in insulin sensitivity index (ISI) were found between two groups, then the HFD group received streptozotocin (35 mg/kg, i.p.); **✓** = improve/restore/regenerate/repair; ↑ = increase/elevate/upregulate; ↓ = decrease/reduce/attenuate/downregulate; O = no difference/no effects; X = blocked/inhibit/prevent; ACE1 = angiotensin converting enzyme 1; BUN = blood urea nitrogen; CAT = catalase; CIPN = chemotherapy-induced peripheral neuropathy; DPN = diabetic painful neuropathy; DRG = dorsal root ganglion; FBG = fasting blood glucose; GSH-Px or GPx = glutathione peroxidase; GSH; = glutathione; H_2_O_2_ = hydrogen peroxide; HDL-C = high-density lipoprotein cholesterol; IL-1β = interleukin-1β; IL-6 = interleukin 6; iNOS = inducible nitric oxide synthase; i.p. = intraperitoneal; LC3B = light chain 3B; LDL-C = low-density lipoprotein cholesterol; LPO = lipid peroxidation; MDA = malondialdehyde; MNCV = motor nerve conduction velocity; mRNA = messenger RNA; NADPH = nicotinamide adenine dinucleotide phosphate reduced form; NF-κB = nuclear factor kappa B; NO = nitric oxide; Nrf2 = nuclear factor erythroid 2–related factor 2; OSI = oxidative stress index; SFI = sciatic functional index; SNCV = sensitive nerve conduction velocity; SOD = superoxide dismutase; TAS = total antioxidant status; TG = triglycerides; TNF-α = tumor necrosis factor alpha; TOS = total oxidant status.

**Table 3:** Effects of curcumin on sciatic nerve chronic constriction injury (CCI).

Animals (Sex, Strain)	Dose (mg/kg), Route of Administration, Duration of Treatment	Effects		Reference
		Behavioral Evaluation/Other Diabetic	Histopathological/ Biochemical/Molecular	
Male Sprague-Dawley rats	CCI + curcumin (100 mg/kg, peritoneal, 14 days)	**✓** Mechanical allodynia (von Frey) and thermal hyperalgesia (Hargreaves test)	**X** Immunohistochemical and protein expressions of NMDAR-NR1 in spinal cord and DRG	[133]
Male Sprague-Dawley rats	CCI + curcumin (100 mg/kg, i.p., 14 days)	↑ Thermal withdrawal latency (heat stimulus) 7 days after surgery and mechanical withdrawal threshold (von Frey) 10 days after surgery	↓ NF-κB p65 protein expression in lumbar spinal cord and DRG 7 days after surgery ↓ CX3CR1 positive expression in spinal dorsal horn and DRG 7 days after surgery	[131]
Male Sprague-Dawley rats	CCI + curcumin (100 mg/kg, peritoneal, 14 days)	**✓** Thermal hyperalgesia (Hargreaves test) and paw withdrawal mechanical threshold (von Frey) on day 14	↓ Serum cortisol concentration **X** Upregulated expression of 11βHSD1 in spinal dorsal horn and DRG	[132]
Male Wistar rats	CCI + curcumin (12.5, 25 and 50 mg/kg, i.p., 7 days)	**O** Mechanical allodynia (von Frey) High dose ↓ Cold allodynia (acetone test)	High dose ↓ Serum concentration of cyclooxygenase 2	[137]
Male Sprague-Dawley rats	CCI + curcumin (20, 40, or 60 mg/kg, i.p., 14 days)	↓ Thermal hyperalgesia (Hargreaves test) and mechanical allodynia (von Frey)	↓ Recruitment of p300/CBP and acetyl-histone H3/acetyl-histone H4 to the promoter of BDNF and Cox-2 genes ↓ mRNA and protein expressions of BDNF and Cox-2 in spinal cord	[135]
Male Sprague-Dawley rats	CCI + curcumin (50 mg/kg, oral, 7 days)	↓ Mechanical allodynia (von Frey)	**O** Protein expressions of p-ERK, p-JNK, and p-NR1 in DRG	[136]
Male C57BL/6J mice	CCI + curcumin (5, 15 or 45 mg/kg, oral, twice per day for 3 weeks)	Chronic treatment ↓Thermal hyperalgesia (hotplate) and mechanical allodynia (von Frey) Depletion of descending noradrenaline (NA) by 6-OHDA **X** Mechanical allodynia, but not thermal hyperalgesia Depletion of 5-HT by PCPA **X** thermal hyperalgesia but not mechanical allodynia Antagonists β-AR (propranolol) but not α-AR (phentolamine) **X** Mechanical allodynia β2-AR (ICI 118,551) **X** Mechanical allodynia 5-HT1A (WAY-100635) **X** Thermal hyperalgesia Antagonists Delta-opioid (naltrindole hydrochloride) ↓ Mechanical allodynia Mu-opioid (β-funaltrexamine) **X** Thermal hyperalgesia Kappa-opioid (nor-binaltorphimine) **O** Mechanical allodynia or thermal hyperalgesia	Chronic treatment increased spinal monoamine serotonin and its metabolite MHPG Did not alter other monoamines/ metabolites (NA, 5-HIAA, dopamine and DOPAC)	[134]
**Combination Study**				
Male Sprague-Dawley rats	CCI + curcumin (100 mg/kg, oral, 14 days) CCI + tramadol (10 mg/kg, i.p., 14 days) CCI + chronic constriction release (CCR) + curcumin (100 mg/kg, oral, 14 days) CCI + CCR + tramadol (10 mg/kg, i.p., 14 days) CCI + curcumin (100 mg/kg, oral, 14 days) + tramadol (10 mg/kg, i.p., 14 days)	CCI + tramadol, and CCI + CCR + tramadol ↑ Thermal hyperalgesia (heat stimulus) CCI + tramadol, and CCI + CCR + tramadol ↑ Mechanical allodynia (dynamic plantar test) **O** Cold-induced pain (cold plate test)	↓ Sciatic and DRG TNF-α in CCI + CCR + tramadol ↑ Sciatic IL-10 in CCI + CCR + tramadol, whereas ↑ DRG IL-10 in CCI + tramadol followed by CCI + CCR + tramadol ↑ Number of regenerated axons in CCI + CCR + curcumin and CCI + CCR + tramadol	[144]

Behavioral modalities are mentioned within parentheses. **✓** = improve; **✓** = improve; ↓ = decrease/reduce; O = no effects; X = abolish/abrogate/block; BDNF = brain-derived neurotrophic factor; 11βHSD1 = 11β-hydroxysteroid dehydrogenase type I enzyme; CBP = CREB-binding protein; CCI = chronic constriction injury; CCR = chronic constriction release; Cox-2 = cyclooxygenase-2; CX3CR1 = CX3C chemokine receptor 1; DRG = dorsal root ganglion; i.p. = intraperitoneal; mRNA = messenger RNA; NF-κB = nuclear factor kappa B; NMDAR NR1 = N-methyl-d-aspartate receptor subunit NR1; TNF-α = tumor necrosis factor alpha.

**Table 4:** Effects of curcumin on sciatic nerve crush injury (SNC), spared nerve injury (SNI), and sciatic nerve ligation (SNL).

Animals (Sex, Strain)	Dose (mg/kg), Route of Administration, Duration of Treatment	Effects			Reference
		Behavioral/Other	Electrophysiological/ Functional	Histopathological/ Biochemical/Molecular	
**Sciatic Nerve Crush (SNC) Injury**					
Male Sprague-Dawley rats	SNC + curcumin dissolved in polyethylene glycol 300 at a concentration of 0.035 mg/µL, 0.2 mg/day osmotic minipump infusion, 28 days	**✓** Mechanical (von Frey), finger spacing of injured paw (visual static sciatic index), skillful walking (beam walking task test), grip strength (grip strength test)	↑ MNCV and SNCV	↑ Myelin sheath thickness ↑ MPZ and PMP22 expressions ↓ Neurogenic lesions ↓ Macrophage-induced production of ROS, lipid peroxidation ↑ Transcription factor Nrf2 expression	[150]
Female Wistar Albino rats	SNC + curcumin (100 mg/kg, nasogastric tube, 28 days)	Not tested	↑ SFI values **✓** Amplitude values and latency time O Gastrocnemius muscle weight	**O** G ratios **✓** Myelin thickness, axon diameter, and nerve diameter	[154]
Male Sprague-Dawley rats	SNC + curcumin (50, 100, 300 mg/kg, i.p., 4 weeks)	↑ Mechanical withdrawal threshold (von Frey) ↓ Thermal withdrawal threshold (hotplate)	↑ SFI values **✓** NCV, CMAP latency onset and peak amplitude ↓ Atrophy of gastrocnemius muscle	↑ Number of fluoro-gold-positive neurons ↑ Number of myelinated axons per nerve transverse section and mean diameter of nerve fibers	[152]
Female Sprague-Dawley rats	SNC + curcumin (100 mg/kg, gavage, 28 days)	Not tested	Not tested	↓ Decreased volume of ganglion, mean cell volume, total volume of DRG cells (A- and B-cells), total surface of DRG cells, total number, diameter, and area of myelinated nerve fibers	[153]
**Combination Study**					
Male Wistar rats	SNC + curcumin (100 mg/kg, i.p., 4 weeks) + melatonin (10 mg/kg, i.p., 4 weeks) during light (9 am) and dark (9 pm) periods	Not tested	Light and dark curcumin **✓** SFI Dark melatonin group **✓** SFI Light and dark curcumin **O** Amplitudes and conduction latencies of evoked CMAP recorded in gastrocnemius muscle Curcumin **✓✓** Shortest latency and greatest amplitude Light and dark curcumin **✓✓** Smallest gastrocnemius muscle atrophy Dark melatonin **✓✓** Gastrocnemius muscle mass	Light and dark curcumin **✓✓** Lesser TOS Light and dark curcumin **✓✓** Higher color intensity of nerve myelin staining (Luxol Fast Blue staining) Dark melatonin **✓✓** Higher nerve myelin staining Light and dark curcumin **✓✓** Higher number of Schwann cells Light curcumin **✓✓** Increased number of neurofilament-positive stained areas Dark melatonin **✓✓** Better neurofilament-positive stained areas	[151]
**Spared Nerve Injury (SNI)**					
Male Sprague-Dawley rats	SNI + curcumin (100 mg/kg, i.p., 4 weeks) SNI + PI3K inhibitor LY294002 (30 mg/kg, i.p., 10 min before curcumin administration) + curcumin (100 mg/kg, i.p., 4 weeks) SNI + siNGF (1ng in 5µL, i.t., 10 min before curcumin administration) + curcumin (100 mg/kg, i.p., 4 weeks) In vitro: 30 mM	Not tested	Not tested	**✓** PC-12 neurons against H_2_O_2_-induced apoptosis by ↑ TrkA, Akt and ↓ p17 ↓ pro-NGF but ↑ mature NGF level PI3K/Akt inhibition ↑ Apoptotic rate by decreasing p17, Ki67, and cyclin D1 NGF suppression and PI3K inhibition ↑ Neuron cell death by increasing proNGF and decreasing mNGF, Akt, TrkA, p75NTR, and p17	[158]
Male BALB/c mice	SNI or sham + curcumin (30, 60, 120 mg/kg, i.p., twice daily from day 1 until day 7 after surgery)	↓ Mechanical (von Frey) and cold (acetone test) allodynia	Not tested	**X** IL-1β protein level **X** NALP1 inflammasome aggregation and JAK2-STAT3 cascade activation in astrocytes	[159]
**Spinal Nerve Ligation (SNL)**					
Female Wistar rats	SNL or sham + curcumin (30, 100, 200, and 300 μg, i.t., 14 days after surgery) SNL or sham + curcumin (10, 100, 310 mg/kg, oral, 14 days after surgery)	**X** SNL-induced mechanical allodynia (von Frey) Curcumin + Inhibitors-NO synthase (L-NAME), guanylyl cyclase (ODQ), and ATP-sensitive K+ channel (glibenclamide) **X** anti-allodynic activity	Not tested		[160]
Sprague-Dawley rats, sex not specified	SNL + curcumin (200 μg, i.t., 7th, 8th, 9th, 10th, 15th, and 20th day after surgery)	↓ SNL-induced mechanical allodynia (von Frey) from 10th day post-treatment	Not tested	Not tested	[161]

Behavioral modalities are mentioned within parentheses. **✓** = protect/improved; **✓✓** = exert/induce; ↑ = increase/upregulate; ↓ = decrease/downregulate; O = no difference; X = prevented/inhibited; CMAP = compound muscle action potential; DRG = dorsal root ganglion; H_2_O_2_ = hydrogen peroxide; IL-1β = interleukin-1β; i.p. = intraperitoneal; i.t. = intrathecal; JAK2-STAT3 = Janus kinase 2-signal transducer and activator of transcription 3; MNCV = motor nerve conduction velocity; MPZ = myelin protein zero; NALP1 = NAcht leucine-rich-repeat protein 1; NCV = nerve conduction velocity; NGF = nerve growth factor; Nrf2 = nuclear factor erythroid 2–related factor 2; PI3K/Akt = phosphatidylinositol 3-kinase/Akt protein kinase B; PMP22 = peripheral myelin protein 22; ROS = reactive oxygen species; SFI = sciatic functional index; SNC = sciatic nerve crush; SNCV = sensitive nerve conduction velocity; SNI = spared nerve injury; SNL = spinal nerve ligation; TrkA = tropomyosin receptor kinase A.

**Table 5:** Effects of curcumin on postoperative and preemptive analgesia.

Animals (Sex, Strain)	Dose (mg/kg), Route of Administration, Duration of Treatment	Effects		Reference
		Behavioral/Other	Histopathological/ Biochemical/Molecular	
**Postoperative Pain**				
Male Sprague-Dawley rats	Incision + curcumin (0.01, 0.03, or 0.1 mg, i.t.)	↓ Mechanical hypersensitivity (von Frey) Antagonists GABA-A (bicuculline) and GABA-B (saclofen) **X** antinociceptive activity Antagonists mu (CTOP), delta (naltrindole), and kappa (GNTI) opioid receptor **X** antinociceptive activity	↑ mRNA expressions of GABA-A and GABA-B in incised spinal cord **O** mRNA expressions of opioid receptors in incised spinal cord	[173]
Male C57BL/6 mice	Incision + curcumin (50 mg/kg, i.p., 4 days)	↓ Mechanical hypersensitivity (von Frey) ↓ Thermal hypersensitivity (Hargreaves test) ↓ Prostaglandin-induced hyperalgesic priming **O** Paw edema (laser sensor technique) and hindpaw temperature (fine wire thermocouple) **O** Morphine-induced place preference (affective component of incision measured by conditioned CPP) **X** Functional abnormalities in gait indices (gait analysis)	**O** IL-1β, IL-6, macrophage inflammatory protein-1α at peri-incisional level **O** IL-10 at peri-incisional level ↑ TGF-β levels at peri-incisional level	[171]
Male Sprague-Dawley rats	Acute treatment: Incision + curcumin (10–40 mg/kg, oral, 1 day after surgery) Repeated treatment: Incision + curcumin (10–40 mg/kg, oral, 20 min before to surgery and twice daily for 7 days) Repeated treatment before surgery: Curcumin (10–40 mg/kg, oral, twice daily for 7 days before surgery) + incision	Acute treatment ↓ Mechanical hyperalgesia (von Frey) Repeated treatment before surgery **O** Mechanical hyperalgesia Repeated treatment **✓** Mechanical hyperalgesia Repeated treatment **✓** Recovery from surgery Repeated treatment before surgery **O** recovery rate **O** Locomotor activity (YLS-1B apparatus)	Not tested	[170]
**Preemptive Analgesia**				
Common crossbred swine	Curcumin [(130 mg/kg, oral, 3 days prior to CPB and extracorporeal support surgery	Not tested	↓ Concentrations of IL-6, TNF-α, and ICAM-1	[174]
Male Wistar Bratislava Albino rats	Nitroglycerin (NTG) (1 mg/100 g body weight i.p.) + curcumin (10 mg/100 g body weight, i.p., 14 days before NTG administration)	↓ Number of flinches and shakes (formalin test)	↓ Blood pressure ↓ MDA, NO, TOS, and thiol compound ↑ TAC	[175]
Female Wistar Albino rats	Curcumin (400 mg/kg, oral, 45 min before formalin injection)	↓ Thermal pain (hotplate test) ↓ Number of flinches (formalin test)	Not tested	[176]

Behavioral modalities are mentioned within parentheses. **✓** = improve, facilitate; ↓ = alleviate/decrease/attenuate; O = no effects/no alteration; X = abrogate/prevent; CPB = cardiopulmonary bypass; CPP = conditioned placed preference; GABA = gamma-Aminobutyric acid; ICAM-1 = intercellular adhesion molecule 1; IL-1β = interleukin-1β; IL-6 = interleukin 6; i.p. = intraperitoneal; i.t. = intrathecal; MDA = malondialdehyde; mRNA = messenger RNA; TAC = total antioxidative capacity; TGF-β = transforming growth factorβ; TNF-α = tumor necrosis factor alpha; TOS = total oxidative status.

**Table 6:** Effects of different curcumin formulations on neuropathic pain.

Animals (Sex, Strain)	Dose (mg/kg), Route of Administration, Duration of Treatment	Effects			Reference
		Behavioral Evaluation/ Other Parameters	Electrophysiological/ Functional Evaluation	Histopathological/ Biochemical/Molecular Parameters	
**Diabetic Neuropathy**					
**Curcumin Derivative**					
Female Swiss Webster mice or diabetic rats (strain and sex were not specified)	STZ − 90 mg/kg, i.p. STZ + phenyl hydrazide derivative J147 (10, 50 mg/kg, i.p. oral, 20 weeks)	↓ Blood glucose and HbA1c levels ↑ Paw thermal response (Hargreaves test) ↓ Tactile allodynia (von Frey) **O** Sensorimotor function (rotarod test)	↓ MNCV	↓ TNFR1, TNFR2, and type I diabetes mellitus signaling pathways ↑ AMPK, and ephrin receptor signaling pathways ↓ Protein levels of TNF-α, TSPO, iNOS or GFAP and peripheral inflammation marker C-reactive protein	[27]
Male SPF rats	STZ − 50 mg/kg, i.p. STZ + J147 (10 or 100 μM of at 10 mg/kg weight, 5 days) In vitro: J147 (10 and 100 μM)	↓ Mechanical withdrawal threshold (von Frey)		**O** Cell viability and apoptosis of RSC96 cells ↑ AMPK mRNA and protein expression levels ↓ TRPA1 mRNA and protein expression levels ↓ Calcium reaction level in AITCR treated RSC96 cells	[33]
**Nanoparticle-Encapsulated Curcumin**					
Male Sprague-Dawley	STZ − 30 mg/kg, i.p. STZ + nanoparticle-encapsulated curcumin, 16 mg/kg, sublingual vein, 7th, and 8th week	↓ Mechanical (electronic mechanical stimulator) and thermal (thermal paw stimulator) hyperalgesia		Interacted perfectly with P2Y12 receptor agonist-binding pocket ↓ mRNA and protein expressions of P2Y12 in DRG ↓ Co-localization of glutamine synthetase (a marker of SGCs) in DRG ↓ mRNA and protein expression of IL-1β and Cx43 expressions in DRG **X** AKT activation	[28]
**Self-Nano Emulsifying Drug Delivery System (SNEDDS) Curcumin**					
Male Sprague–Dawley rats	STZ − 55 mg/kg, i.p. STZ + naïve curcumin (30, 100 and 300 mg/kg, oral, 2 weeks) STZ + SNEDDS curcumin (30, 100 and 300 mg/kg, oral, 2 weeks)	**O** Body weight and plasma glucose level **✓** Thermal hyperalgesia (tail flick test) in both hot and cold immersion **✓** Mechanical hyperalgesia (von Frey and Randall Sellitto tests)	Naïve and SNEDDS ↓ MNCV and NBF	↓ MDA levels SNEDDS ↓ NF-κB protein expression SNEDDS **X** IKK-β phosphorylation expression SNEDDS ↓ Protein expression of NF-κB positive cells in nerves SNEDDS ↓ COX-2 and iNOS protein level Naïve and SNEDDS ↓ IL-6 level in sciatic nerves SNEDDS ↓ TNF-a level in sciatic nerves	[30]
**CCI**					
**Curcumin (Cur) Loaded with Lipid Nanocapsules (Cur@LNCs)**					
Female Sprague-Dawley rats	CCI + Cur@LNCs, 400 µL inject, 7 days	↓ Thermal hyperalgesia (hotplate)		↓ Sciatic nerve damages	[29]
**Curcumin Prodrug-Curcumin Diglutaric Acid**					
Male ICR mice	CCI + curcumin diglutaric acid (CurDG) (25, 50, 100, and 200 mg/kg, oral, 14 days)	**✓** Mechanical allodynia (von Frey), and thermal hyperalgesia (plantar test) **O** Motor performance (rotarod test)		↓ Overexpression of TNF-α and IL-6 levels in both sciatic nerve and spinal cord	[32]
**Curcumin-Loaded Poly (d, l-lactide-co-glycolide) Nanovesicles**					
Male CD1 mice	CCI + curcumin (20 mg/kg, intravenous or 0.0005 and 0.025 mg, i.t.) CCI + PLGA CUR (0.045 mg curcumin/mg of nanoparticles, 20 mg/kg, intravenous) CCI + PLGA CUR (0.045 mg curcumin/mg of nanoparticles, 0.0005 and 0.025 mg, i.t.)	Low and high PLGA-CUR, i.t. ↓ Mechanical allodynia (dynamic plantar aesthesiometer test) and thermal hyperalgesia (plantar test) High curcumin, i.t. ↓ allodynia and hyperalgesia		High PLGA-CUR, i.t. ↓ IL-1β, IL-6,TNF-α and BDNF levels in spinal cord	[34]
**Curcumin Derivative**					
Male ICR mice	Curcumin derivative KMS4034 (10 mg/kg, i.p., 120 min post-injection) In vitro: 10 µM KMS4034	↑ Mechanical thresholds (von Frey)	**X** ICAP and Iheat of TRPV1-expressing HEK293 cells	↓ CGRP expression in lamina I–II of lumbar dorsal horns	[31]

Behavioral modalities are mentioned within parentheses. **✓** = improve; ↑ = increase; ↓ = diminish/decrease/ameliorate; O = no effects/no changes; X = block/inhibit; AMPK = AMP-activated protein kinase; BDNF = brain-derived neurotrophic factor; Cox-2 = cyclooxygenase-2; Cur@LNCs = curcumin (Cur) loaded with lipid nanocapsules; Cx43 = connexin 43; DRG = dorsal root ganglion; GFAP = glial fibrillary acidic protein; ICR = institute of cancer research; IL-1β = interleukin-1β; interleukin 6; iNOS = inducible nitric oxide synthase; i.p. = intraperitoneal; i.t. = intrathecal; MDA = malondialdehyde; mRNA = messenger RNA; P2Y12 = purinergic receptor 12; PLGA-CUR = curcumin-loaded poly (d, l-lactide-co-glycolide) nanovesicles; SGC = satellite glial cells; SNEDDS = self-nano emulsifying drug delivery system; TNF-α = tumor necrosis factor alpha; NF-κB = nuclear factor kappa B; TNFR1 = tumor necrosis factor alpha receptor 1; TNFR2 = tumor necrosis factor alpha receptor 2; TSPO = translocator protein.

**Table 7:** Effects of curcumin on neuropathic pain and postoperative pain of clinical studies.

Participants and Study Design	Dose and Duration	Effects		Reference
		Pain-Related	Other/Cardiometabolic	
**Diabetic Sensorimotor Polyneuropathy (DSPN)**				
Patients with T2D (*n* = 80); RCT (placebo-controlled and double-blind)	Nano curcumin (72% curcumin, 80 mg) or placebo capsules/day for eight weeks	↓ Score of total neuropathies, reflex score, and temperature in curcumin vs. placebo	↓ HbA1c and FBS in curcumin vs. placebo	[211]
**Chemotherapy-Induced Peripheral Neuropathy (CIPN)**				
Patients undergoing cancer chemo- and radiotherapy (*n* = 160); RCT (placebo-controlled and double-blind)	Lecithinized curcumin (Meriva: 500 mg) or placebo for 60 days from first cycle of chemo- or radiotherapy	↓ Local pain rating based on VAS due to radiotherapy in curcumin vs. placebo group	↓ Chemotherapy side effects in curcumin vs. placebo group	[212]
**Peripheral Neuropathy (PN)**				
Patients with chronic PN and lumbar disc herniation and/or lumbar canal stenosis or carpal tunnel syndrome; (*n* = 135); RCT, open	Three formulations as follows: (1) Dex (800 mg) + Lipicur [lipoic acid (800 mg) + curcumin phytosome (800 mg) + piperine (8 mg)]; (2) Dex + lipoic acid (800 mg) and (3) Dex only (800 mg) capsules/day for eight weeks	↓ Neuropathic pain in patients with lumbar sciatica and carpal tunnel syndrome in Lipicur group vs. others	↓ Use of Dex in the Lipicur group vs. others	[215]
**Postoperative Pain**				
Patients undergoing oral surgery for periodontitis (*n* = 15); RCT (placebo-controlled)	Curcumin mucoadhesive film (0.5% extract) or placebo mucoadhesive film placed on gingiva after surgery for seven days	↓ Pain score rating and swelling in curcumin vs. placebo group	↓ Use of oral analgesics in postoperative period in curcumin vs. placebo group	[210]
Patients following laparoscopic gynecologic surgery (*n* = 60); RCT, open	Curcuminoids extract (1000 mg) or standard analgesia on postoperative days one to three	↓ VAS pain scores following surgery in curcumin vs. standard group	N/A	[214]
Patients undergoing oral surgery for impacted third molars (*n* = 90); RCT (placebo-controlled)	Curcumin (200 mg) + amoxicillin (500 mg) or control (amoxicillin 500 mg + 500 mg mefenamic acid) three times for 24 h	↓ Pain score rating in curcumin vs. placebo group	N/A	[213]
Patients undergoing laparoscopic cholecystectomy (*n* = 50); RCT (placebo-controlled and double-blind)	Curcumin (500 mg) or placebo once every six hours/day for three weeks	↓ Pain score rating in curcumin vs. placebo group	↓ Fatigue score and the use of oral analgesics in postoperative period in curcumin vs. placebo group	[209]

↓ = Decrease/lower; CIPN = chemotherapy-induced peripheral neuropathy; Dex = dexibuprofen; DSPN = diabetic sensorimotor polyneuropathy; FBS = fasting blood glucose; HbA1c = glycated hemoglobin; N/A = not applicable; PN = peripheral neuropathy; RCT = randomized controlled trial; T2D = type 2 diabetes; VAS = visual analog scale.

## Data Availability

Not applicable.
